# Can Clinical, Psychophysical or Psychological Variables Help in Discriminating Women with Migraines from a Tertiary Center? A Diagnostic Accuracy Study

**DOI:** 10.3390/diagnostics14242805

**Published:** 2024-12-13

**Authors:** Margarita Cigarán-Mendez, Juan C. Pacho-Hernández, Francisco G. Fernández-Palacios, Ángela Tejera-Alonso, Juan A. Valera-Calero, Cristina Gómez-Calero, Carlos Ordás-Bandera, César Fernández-de-las-Peñas

**Affiliations:** 1 Department of Psychology, Universidad Rey Juan Carlos, 28922 Alcorcón, Spain; margarita.cigaran@urjc.es (M.C.-M.); juancarlos.pacho@urjc.es (J.C.P.-H.); gines.fernandez@urjc.es (F.G.F.-P.);; 2 Escuela Internacional de Doctorado, Universidad Rey Juan Carlos, 28922 Alcorcón, Spain; 3 Department of Radiology, Rehabilitation and Physiotherapy, Faculty of Nursery, Physiotherapy and Podiatry, Complutense University of Madrid, 28040 Madrid, Spain; juavaler@ucm.es; 4 Grupo InPhysio, Instituto de Investigación Sanitaria del Hospital Clínico San Carlos (IdISSC), 28040 Madrid, Spain; 5 Department Physical Therapy, Occupational Therapy, Rehabilitation, and Physical Medicine, Universidad Rey Juan Carlos, 28922 Alcorcón, Spain; cristina.gomez@urjc.es; 6 Department of Neurology, Hospital Rey Juan Carlos, 28933 Móstoles, Spain; ordas.carlos@gmail.com

**Keywords:** migraine, diagnostic accuracy, pressure pain, burden, anxiety, depression

## Abstract

**Background:** Migraine diagnosis is mainly clinically based on symptomatology. The objectives of this study were (1) to determine the ability of pain thresholds to differentiate between women with and without migraines and (2) to determine the ability of clinical, psychological and psychophysical variables to differentiate between women with episodic and chronic migraines. A diagnostic accuracy study was conducted. **Methods:** Pressure-pain thresholds (PPTs) at one trigeminal (temporalis muscle) and one extra-trigeminal (cervical spine) and two distant-pain free (second metacarpal and tibialis anterior muscle) areas, as well as dynamic pain thresholds (DPTs), were bilaterally assessed in 100 women with migraines, recruited from tertiary hospitals (50% episodic, 50% chronic), and 50 comparable women without headaches. Migraine pain features (headache diary), migraine-associated burden (HDI), anxiety and depressive levels (HADS) and state (STAI-S)–trait (STAI-T) anxiety were also evaluated. The area under the receiver operating characteristic (ROC) curve, with optimal cut-off points, as well as the sensitivity, specificity and positive/negative likelihood ratios (LR) for each variable, were calculated. The women with migraines showed lower PPTs and DPTs than those without migraines. **Results:** The women with chronic migraines showed lower PPTs in the temporalis muscle than the women with episodic migraines. No clinical, psychological or psychophysical variables exhibited acceptable ROC values (≥0.7) for differentiating between women with and without migraines or between women with episodic and chronic migraines. **Conclusions:** Although the women with migraines had widespread pressure-pain hyperalgesia, neither the clinical, psychological nor psychophysical (pain threshold) variable exhibited the proper diagnostic accuracy to distinguish between women with and without migraines or between women with episodic and chronic migraines. New studies should clarify the clinical relevance of the findings of the current study.

## 1. Introduction

Migraines are a primary headache disorder, commonly seen in clinical practice, that is associated with substantial burden [1]. Thus, the Global Burden Neurological Disease Study found that migraine headache was the third-ranked disorder in terms of absolute number of years lived with disability (DALYs) in the United States of America [2]. The estimated worldwide prevalence of migraines has been reported to be 14.0% (95% CI: 12.9–15.2) [3].

Although the episodic form of migraines is the most prevalent clinical presentation, about 2.5% of individuals progress to the chronic form [4]. Different hypotheses have been proposed for explaining migraines: vascular theory (i.e., the activation of trigemino-vascular pathways), brainstem theory (i.e., brain dysfunction with deficient regulation of cortical activity) and sensitization theory (i.e., altered nociceptive processing at the trigemino-cervical nucleus caudalis) [5].

Altered nociceptive processing has been manifested by pain hyperalgesia in response to different stimuli using quantitative sensory testing [6]. In the headache literature, pressure, electrical and thermal stimuli are the quantitative sensory tests most used for evaluating the presence of altered nociceptive processing [7]. Evidence supports the presence of pressure-pain hyperalgesia, as expressed by lower pressure-pain thresholds (PPTs), as a consistent finding in patients with migraines [8] Thus, there is also evidence supporting that migraine sufferers also exhibit thermal and electrical pain hyperalgesia, although these findings are less consistent [9]. Both meta-analyses concluded that altered nociceptive processing observed in individuals with migraines is modality-, measure- and location-specific [8,9]. In fact, those authors asked for the identification of the most appropriate quantitative sensory tests that were able to differentiate between patients with migraines and controls without headaches.

In addition to biological factors, psychological factors and stress can also play important roles in the precipitation of migraines. In fact, stress was the main trigger factor for migraines in up to 58% of patients [10]. Thus, depressive and anxiety symptoms are comorbidities observed in almost 25% of patients with migraines [11].

Interestingly, although it seems clear that patients with migraine with altered nociceptive pain processing have lower PPTs and those with mood disorders have higher levels of anxiety and depression than controls without headaches, no study has investigated if hyperalgesia in response to pressure pain or other clinical variables could help in the diagnosis of this condition. Due to the presence of sex differences in the prevalence of migraines, i.e., a 2:1 female/male ratio [3], and in pain sensitivity, i.e., women with migraines exhibiting lower PPTs than men [8], we included only women in the current study. Accordingly, the aims of the current study were (1) to determine the capability of pressure-pain thresholds to differentiate between women with and without migraines and (2) to assess the capability of clinical, psychological and psychophysical variables to differentiate women with episodic migraines from women with chronic migraines. We analyzed the area under the receiver operating characteristic (ROC) curve, the optimal cut-off point, the sensitivity, the specificity and the positive and negative likelihood ratio (LR) for each variable.

## 2. Methods

### 2.1. Participants

Women diagnosed with migraines, as defined by the International Headache Society third-edition criteria [12], who attended a tertiary hospital were consecutively recruited by an experienced neurologist. The migraine history documentation included details such as the location of pain attacks, the quality of the pain, the onset of the migraines (years), the frequency and severity of the attacks, family history of migraines and medication usage.

For the control group, women with no prior migraine diagnosis or reporting no headaches in the past year were recruited through local advertisements. These controls without headaches were matched by age to the migraine group. All participants underwent a clinical examination by a neurologist to confirm their eligibility for this study.

The exclusion criteria included the following conditions: (1) other types of primary or secondary headache, including medication overuse headache [12]; (2) a history of head or neck trauma (e.g., whiplash); (3) pregnancy; (4) a cervical herniated disc; (5) other medical comorbidities, such as rheumatoid arthritis; (6) other chronic pain conditions, such as cancer pain or fibromyalgia; or (7) any recent medical intervention in the past three months, including aesthetic block treatments.

All subjects signed the informed consent form before their inclusion in this study. The Ethics Committee of Hospital Rey Juan Carlos (HRJC 07/14) approved this study.

A headache diary was used to collect the following data: the frequency of migraines (days per month), the duration of migraine attacks (hours per attack) and the intensity of the attacks (Numerical Pain-Rating Scale [NPRS], 0–10 points) [13]. Further, the Headache Disability Inventory (HDI) was used to evaluate the physical (HDI-P, 12 items, 0–48 points) and emotional (HDI-E, 13 items, 0–52 points) burdens associated with migraines [14]. The HDI has shown excellent reliability, with coefficients ranging from 0.76 to 0.83 [15]. 

### 2.2. Quantitative Sensory Tests: Mechanical Pain Thresholds 

In this study, static and dynamic sensitivity to pressure pain were assessed by a clinician blinded to the subject condition. In the women with episodic migraines, all pain assessments were conducted in a headache-free status of the patients and after at least one week since the last migraine attack (to avoid migraine-related allodynia). In the women with chronic or quasi-daily chronic migraines, all pain assessments were conducted pain-free (if possible) or when the intensity of the headache attack was <3 points. Further, in these patients, we also tried to conduct these assessments after at least one to two days since the last migraine attack. In addition, the patients were also asked to avoid the intake of any analgesic or muscle relaxant for at least 24 h prior to the quantitative sensory pain assessment. Thus, prophylactic treatment was not changed at all. 

For evaluating widespread pain sensitivity, PPTs were bilaterally assessed over the temporalis muscle (i.e., the trigeminal area), the C5/C6 zygapophyseal joint (i.e., the extra-trigeminal area) and the second metacarpal and tibialis anterior muscle (i.e., the pain-free distant areas) with an electronic algometer (Somedic AB^®^, Farsta, Sweden). Each threshold was repeated thrice on each point, and the mean was calculated and used in the statistical analysis. A 30 s resting period was allowed between every two trials to avoid temporal summation [16]. The order of assessment between each point was randomized. Pressure algometry has shown moderate to high reliability, with coefficients ranging from 0.55 to 0.78 [17].

For dynamic pain sensitivity, dynamic pressure thresholds (DPTs, defined as the load level of a roller that is first perceived as painful) were bilaterally assessed over the trigeminal area with a specific dynamic pressure algometer (Aalborg University^®^, Aalborg, Denmark), as previously described [18]. The dynamic pressure algometer has eleven different rollers with the following fixed load levels: 500 g, 700 g, 850 g, 1350 g, 1550 g, 2200 g, 2500 g, 3100 g, 3500 g, 3850 g and 5300 g [18]. The procedure was the same as for the PPTs. Dynamic pressure algometry has shown moderate to high reliability, with coefficients ranging from 0.75 to 0.88 [19].

### 2.3. Psychological Variables

The Hospital Anxiety and Depression Scale (HADS) was used to calculate the presence of depressive (HADS-D, 7 items, 0–21 points) and anxious (HADS-A, 7 items, 0–21 points) symptomatology [20]. The HADS has been shown to be a valid and reliable self-reported outcome measure for evaluating anxiety and depression levels separately [21]. The HADS has shown internal consistency and reliability when used in headache patients [22]. We used the Spanish version of the HADS, which has been found to be consistent [23].

The State–Trait Anxiety Inventory (STAI) was administered to evaluate the state (STAI-S, 20 items, 0–60 points) and trait (STAI-T, 20 items, 0–60 points) levels of anxiety [24]. The STAI-S evaluates the presence of anxiety symptomatology, whereas the STAI-T assesses the propensity of a person to experience anxiety and the tendency to perceive stressful situations as threatening [24]. The STAI has shown good internal consistency and high reliability [25]. 

### 2.4. Statistical Analysis

The Statistical Package for the Social Sciences (SPSS) v.29.1.1 (Armonk, NY, USA) for Mac OS was used for the data processing and analyses. All tests were two-tailed, with a significance threshold set at *p* < 0.05. The normal distribution of the continuous variables was assessed with histograms and Shapiro–Wilk tests. Differences in the clinical, psychophysical and psychological variables between the women with and without migraines or between the women with chronic and episodic migraines were analyzed with independent Student *t*-tests. 

Diagnostic accuracy analyses were conducted to (1) first, determine the ability of mechanical pain thresholds to distinguish between women with and without migraines and (2) determine the ability of clinical, psychophysical and psychological variables to distinguish between women with episodic and chronic migraines. These analyses included the area under the receiver operating characteristic (ROC) curve (where ROC values ≥ 0.7 indicated acceptable discrimination) [26], the optimal cut-off point for each variable (the Youden index) [27], sensitivity and specificity, as well as the positive and negative likelihood ratios (LRs). We considered when sensitivity was at least 70% and specificity at least 50% to be acceptable values [28].

## 3. Results

### 3.1. Participants

From 130 potential eligible women with headaches, thirty were excluded for the following reasons: tension-type headaches (*n* = 15), whiplash (*n* = 5), fibromyalgia (*n* = 5), receiving aesthetic blocks in the previous 3 months (*n* = 3) or being pregnant (*n* = 2). Thus, a total of 100 women with migraines (age: 42; SD: 13 years; 50% chronic) and 50 comparable female controls without headaches (mean age: 43; SD: 11 years) were included. 

### 3.2. Subjects with Migraines Versus Controls Without Headaches

Table 1 shows the differences in the PPTs and DPTs between the women with (total sample) and without migraines. Overall, the women with migraines showed lower PPTs in all points as well as lower DPTs compared with the women without headaches (Table 1). 

Table 2 shows the diagnostic accuracy of the PPTs and DPTs in differentiating women with migraines from women without headaches. The ROC values indicate how the PPTs discriminated between both groups (values closer to 1.0 represent better discrimination). As shown in Table 2, neither the PPTs nor DPTs show acceptable ROC values (≥0.7). Overall, the PPTs and DPTs had Youden indices of 0.000, suggesting they do not offer any meaningful diagnostic value in clinical practice.

**Figure 1:**
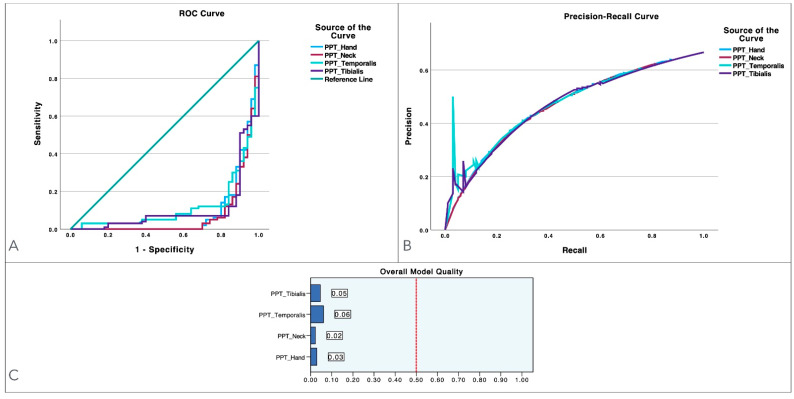
Model performance illustration for pressure-pain thresholds (PPTs) measured at the temporalis, cervical spine, second metacarpal and tibialis anterior muscle, as well as for dynamic pain thresholds (DPTs) in women with/without migraines, using both ROC and precision–recall curves. Panel (**A**) presents the ROC curve, highlighting the sensitivity–specificity trade-off for each variable. Panel (**B**) displays the precision–recall curve, offering additional insight into each variable’s performance. Panel (**C**) consists of bar charts that summarize the overall model quality for each variable.

Figure 1 presents three analyses to assess the predictive model’s performance. In the ROC curve (Figure 1A), none of the thresholds demonstrate effective classification capability. The precision–recall curves in Figure 1B plot precision (true positives as a proportion of the predicted positives) against recall (true positives as a proportion of the actual positives) over various threshold settings. The precision–recall curves emphasize the model’s performance regarding the positive class, providing insight into its accuracy of identifying positive instances while minimizing false positives. However, as shown in Figure 1B, no threshold achieved acceptable precision. Lastly, Figure 1C indicates that none of the models displayed any predictive effectiveness.

### 3.3. Episodic Versus Chronic Migraine 

Table 3 details the comparison of the clinical, psychophysical and psychological variables between the women with episodic and chronic migraines. The women with chronic migraines showed higher headache-associated burden (HDI-E, HDI-P) and lower PPTs in the trigeminal area (temporalis muscle) when compared with the women with episodic migraines (Table 3). No other differences were observed.

Table 4 shows the diagnostic accuracy of clinical and psychological variables for differentiating women with episodic and chronic migraines. Neither the clinical nor psychological headache variables exhibited acceptable ROC values (≥0.7). The only ROC value close to an acceptable cut-off was state anxiety (STAI-T) level (0.587); however, its sensitivity (76%) and specificity (52%) values mean that the presence of higher-state anxiety do not exclude women with episodic migraines. The ROC values of the remaining clinical and psychological variables were small. Thus, most Youden indices were close to 0.000, suggesting they do not offer any meaningful diagnostic value in clinical practice. 

**Figure 2:**
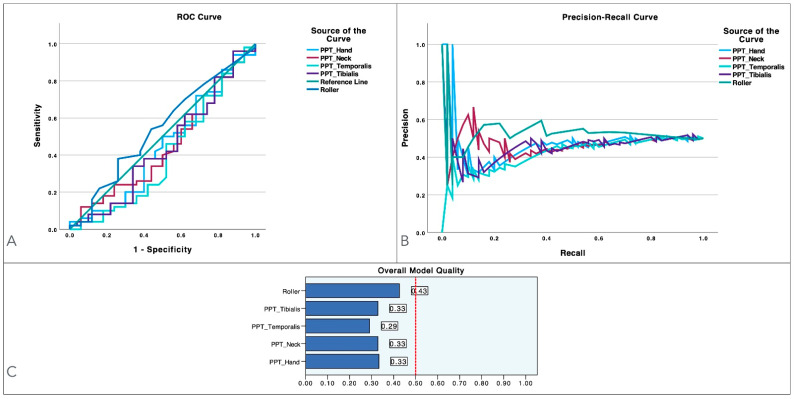
Model performance using ROC and precision–recall curves for migraine-related variables, including migraine intensity, frequency and duration; associated burden (HDI); anxiety (HADS-A); depression (HADS-D); and state (STAI-S) and trait (STAI-T) anxiety levels in women with episodic or chronic migraines. Panel (**A**) presents the ROC curves, depicting the sensitivity–specificity balance for each variable, while Panel (**B**) illustrates the precision–recall curves, offering additional detail on each variable’s performance. Finally, the bar charts in Panel (**C**) summarize the overall model quality for each variable.

Figure 2 graphs the model performance of clinical and psychological variables across different predictors using ROC curves (Figure 2A), PR curves (Figure 2B) and overall model quality (Figure 2C). As illustrated in Figure 2A, all variables showed a good balance of sensitivity and specificity. Figure 2B highlights that no variable exhibited appropriate precision by means of the PR curves. Figure 2C reveals that no variables exhibited appropriate overall model quality (≥0.50). 

Table 5 shows the diagnostic accuracy of the pain thresholds (PPTs and DPTs) for differentiating between women with episodic migraines and chronic migraines. Neither the PPTs nor DPTs showed acceptable ROC values (≥0.7). The highest ROC value was found for the DPTs (0.540); however, the DPTs showed low sensitivity (38%) and low specificity (26%), suggesting that DPTs are not able to exclude women with episodic migraines. Thus, the overall Youden indices were close to 0.000, suggesting that these variables do not offer any meaningful diagnostic value in clinical practice. 

**Figure 3:**
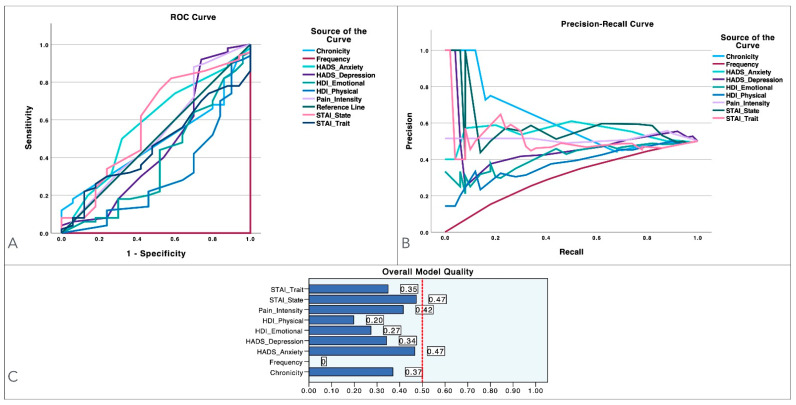
ROC and precision–recall curves for pressure-pain thresholds (PPTs) measured at the temporalis, cervical spine, second metacarpal and tibialis anterior muscle, along with dynamic pain thresholds (DPTs), in women experiencing episodic or chronic migraines. Panel (**A**) shows the ROC curves, illustrating the trade-off between sensitivity and specificity for each variable. Panel (**B**) displays the precision–recall curves, providing further insight into each variable’s performance. Lastly, the bar charts in Panel (**C**) present an overview of the overall model quality for each variable.

Figure 3 graphs the ROC curve (Figure 3A), PR curves (Figure 3B) and overall model quality (Figure 3C) of the pain thresholds. No pain threshold exhibited proper classification ability according to the ROC curves (Figure 3A), appropriate precision provided by the PR curves (Figure 3B) or predictive performance as depicted by the overall model’s quality (Figure 3C).

## 4. Discussion

The results of the current study show that although the women with migraines exhibited hyperalgesia to pressure pain, i.e., widespread lower PPTs and lower DPTs, compared with the women without headaches, no pain threshold exhibited diagnostic accuracy in discriminating between women with and without migraines. Thus, neither clinical, psychological nor psychophysical variables exhibited diagnostic accuracy to differentiate between women with episodic and chronic migraines. 

### 4.1. Differentiating Between Women with and Without Migraines

The current literature supports the presence of widespread pressure-pain hyperalgesia in migraines; however, the results are heterogeneous, since this hyperalgesia in response to pressure pain has been found to be consistent in episodic but not in chronic migraines [8]. This heterogeneity is based on data from single studies, since some found different PPTs in migraine sufferers when compared with controls without headaches [29,30,31,32], whereas others did not find such differences [33,34]. The new finding from this study is that pressure-pain sensitivity was not able to identify women with/without migraines, since the ROC curve of all PPTs did not reach an acceptable value. It is possible that the large inter-group differences between the women with and without migraines could explain this finding, since the identified cut-off points were all over the PPTs observed in the women with migraines. A previous review [35] identified 230 kPa as a potential cut-off score for PPTs at the trigeminal area to determine the presence of sensitization and headaches. Our analysis established a cut-off value of 200 kPa (Table 2) for the trigeminal area, but with an ROC score that was extremely small (0.119). Hence, this cut-off does not provide a meaningful diagnostic value in clinical practice.

The diagnosis of a migraine is a clinical-based diagnosis where the headache features and their associated symptoms are the factors used by neurologists [12]. Interestingly, peri-cranial tenderness is not included in this classification, contrary to what is used for the diagnosis of a tension-type headache (TTH), where the IHS classifies patients with TTHs as associated (codes 2.1.1., 2.2.1., 2.3.1.) or not associated (codes 2.1.2., 2.2.2., 2.3.2.) with tenderness based on manual palpation on the peri-cranial area [12]. For both primary headaches, sensitivity to pressure pain, assessed with an algometer, is not used as a diagnostic tool. Accordingly, our results support that pain sensitivity, as assessed with pressure algometry, does not reveal any diagnostic accuracy in differentiating between women with and without migraines. 

### 4.2. Differentiating Between Episodic and Chronic Migraines

Overall, there were no differences in the PPTs or DPTs between the women with episodic and chronic migraines, except for the PPT at the trigeminal area. These results agree with the findings reported by a recent meta-analysis showing lower PPTs in the trigeminal areas of chronic migraine sufferers than in those with the episodic form [8]. Additionally, the lack of differences in the PPTs in pain-free distant areas supports that widespread sensitivity to pressure pain is similar in the episodic and chronic forms of migraines [8]. This is an unexpected finding, since chronic headache is the most associated with altered nociceptive pain processing, rather than the episodic form. This lack of differences in pressure-pain sensitivity could explain why no pain threshold showed appropriate diagnostic accuracy to differentiate between women with episodic and chronic migraines. Thus, the current results agree with the review by Kamińska et al., which reported that the assessment of pain thresholds is not an approach for the screening and diagnosis of orofacial pain [36]. The previous and current results support the concept that psychophysical outcomes should be considered mechanistic neurophysiological outcomes rather than potential diagnostic tools. 

According to the IHS, the frequency of migraine attacks is the cardinal clinical feature used in classifying patients with episodic (code 1.1) or chronic (code 1.3) migraines [12]. Thus, the cut-off value of attacks differentiating between episodic and chronic migraines is 15 days per month [12]. The frequency of the migraine attacks in our episodic group was clearly different from the chronic group; albeit, this outcome did not exhibit a good diagnostic accuracy score. In fact, no clinical migraine parameter showed an appropriate diagnostic accuracy for differentiating women with episodic and chronic migraines.

Finally, the psychological variables did not show appropriate diagnostic accuracy. Only the state anxiety levels (STAI-T) exhibited an ROC (0.587) close to the cut-off value of 0.7; however, their specificity was small, and the model was poor limiting their potential application in clinical practice. In fact, the presence of anxiety is not exclusive to chronic migraines, since mood disorders are comorbid in most primary headaches [11]. Therefore, the assessment of psychological disturbances in migraines could be used as a complement to clinically related diagnosis if combined with clinical diagnosis.

### 4.3. Limitations 

Although the inclusion of a homogeneous sample of women with episodic or chronic migraines diagnosed by the most updated diagnostic criteria and an exhaustive diagnostic accuracy analysis could be considered the strengths of the current study, some limitations should be recognized. First, we only included women with migraines. It is well-known that women exhibit higher pain sensitivity than men [37], and this has also been found in migraine studies [8]. We do not currently know if similar results would be found in men with migraines. Second, our sample was recruited from an urban tertiary care hospital; therefore, it may be possible that our cohort represents a specific group of the general population with migraines seeking medical treatment. Accordingly, population-based studies including men with migraines are needed to further confirm or refute the current results.

## 5. Conclusions

Women with migraines exhibit pressure-pain hyperalgesia, as expressed by widespread lower PPTs and lower DPTs, when compared with healthy women without headaches. No difference in pressure-pain sensitivity was identified in the women with episodic or chronic migraines, except for in the trigeminal area. No pain threshold showed appropriate diagnostic accuracy to distinguish between women with and without migraines. Thus, neither the clinical, psychological nor psychophysical variables exhibited the diagnostic accuracy to discriminate between women with episodic and chronic migraines. 

## Figures and Tables

**Table 1:** Pressure-pain thresholds (PPTs, means and standard deviations) and dynamic pain thresholds (DPTs, means and standard deviations) in women with (*n* = 100) and without (*n* = 50) migraines.

	Migraine (*n* = 100)	No Migraine (*n* = 50)	*p*
PPT temporalis muscle (kPa)	161.0 (66.5)	275.0 (60.0)	<0.001
PPT cervical spine (kPa)	133.5 (42.0)	236.5 (53.0)	<0.001
PPT second metacarpal (kPa)	194.0 (63.0)	370.0 (96.5)	<0.001
PPT tibialis anterior (kPa)	325.0 (110.5)	520.5 (106.0)	<0.001
DPT temporalis muscle (grams)	960.5 (554.0)	1465.5 (688.5)	0.001

**Table 2:** Diagnostic accuracy analysis and cut-off values of pressure-pain (PPTs) and dynamic pain thresholds (DPTs) to differentiate women with and without migraines.

	PPT—Temporalis	PPT—Cervical Spine	PPT—Hand	PPT—Tibialis
ROC value	0.119	0.072	0.084	0.101
95% CI	0.061; 0.176	0.023; 0.121	0.029; 0.139	0.046; 0.157
Cut-off point	197.6	170.0	313.3	939.0
Youden index	0.000	0.000	0.000	0.000
Sensitivity	0.000	0.000	0.000	0.000
Specificity	0.000	0.000	0.000	0.000
Positive LR	Undefined	Undefined	Undefined	Undefined
Negative LR	Undefined	Undefined	Undefined	Undefined

**Table 3:** Comparison of clinical, psychophysical and psychological variables in women with episodic (*n* = 50) and chronic (*n* = 50) migraines (values are expressed as means and standard deviation).

	Episodic (*n* = 50)	Chronic (*n* = 50)	*p*
Age (years)	41.5 (12.0)	43.0 (13.5)	0.414
Time with headaches (years)	18.3 (13.1)	22.1 (16.4)	0.199
Headache intensity (NPRS, 0–10)	8.3 (1.6)	7.9 (2.1)	0.316
Headache frequency (days/month) #	6.0 (2.4)	22.0 (5.7)	<0.001
Headache duration (hours/attack)	25.0 (22.3)	22.0 (10.0)	0.372
PPT temporalis muscle (kPa) #	175.0 (74.0)	147.5 (55.0)	0.035
PPT cervical spine (kPa)	136.5 (42.5)	130.0 (42.5)	0.423
PPT second metacarpal (kPa)	199.5 (64.0)	188.0 (62.0)	0.353
PPT tibialis anterior (kPa)	337.5 (118.0)	313.0 (102.0)	0.273
DPT temporalis muscle (grams)	962.0 (532.0)	959.0 (581.0)	0.979
HDI-E (0–52) #	25.7 (13.1)	31.9 (14.3)	<0.001
HDI-P (0–48) #	32.7 (11.3)	40.3 (10.8)	0.002
HADS-A (0–21)	12.3 (2.6)	12.0 (2.3)	0.400
HADS-D (0–21)	10.3 (2.5)	10.5 (3.2)	0.733
STAI-S (0–60)	21.7 (4.6)	20.5 (4.2)	0.293
STAI-T (0–60)	25.4 (7.2)	26.1 (6.0)	0.601

NPRS: Numerical Pain-Rating Scale; HDI: Headache Disability Inventory (E: emotional, P: physical); HADS: Hospital Anxiety and Depression Scale (A: anxiety, D: depression); STAI: State–Trait Anxiety Inventory (S: state, T: trait); PPT: pressure-pain threshold; DPT: dynamic pain threshold. # Statistically significant differences.

**Table 4:** Diagnostic accuracy analysis and cut-off values of clinical and psychological variables to differentiate women with episodic and chronic migraines.

	PPT—Temporalis	PPT—Cervical Spine	PPT—Hand	PPT—Tibialis	DPT
ROC Value	0.403	0.442	0.447	0.442	0.540
95% CI	0.290; 0.515	0.328; 0.555	0.333; 0.561	0.328; 0.556	0.427; 0.654
Cut-off Point	159.6	182.7	197.5	359.9	1062.5
Youden Index	0.040	0.060	0.060	0.080	0.120
Sensitivity	0.980	0.120	0.940	0.960	0.380
Specificity	0.940	0.060	0.880	0.880	0.260
Positive LR	16.333	0.128	7.833	8.000	0.514
Negative LR	0.021	1.000	0.068	0.046	2.385

**Table 5:** Diagnostic accuracy analysis and cut-off values of pressure-pain (PPTs) and dynamic pain thresholds (DPTs) to differentiate women with episodic and chronic migraines.

	Migraine Intensity	Migraine Duration	Migraine Frequency	HDI-E	HDI-P	HADS-D	HADS-A	STAI-S	STAI-T
ROC Value	0.530	0.486	0.000	0.384	0.301	0.459	0.580	0.587	0.463
95% CI	0.416; 0.644	0.370; 0.602	0.000; 0.000	0.273; 0.495	0.197; 0.405	0.343; 0.575	0.467; 0.693	0.474; 0.701	0.349; 0.578
Cut-off Point	6.5	20	21	25	30	9.5	12.5	19.5	22.5
Youden Index	0.180	0.120	0.000	0.000	0.000	0.180	0.180	0.240	0.100
Sensitivity	0.880	0.120	0.000	0.000	0.000	0.920	0.500	0.760	0.220
Specificity	0.700	0.000	0.000	0.000	0.000	0.740	0.320	0.520	0.120
Positive LR	2.933	Undefined	Undefined	Undefined	Undefined	3.538	0.735	1.583	0.250
Negative LR	0.171	Undefined	Undefined	Undefined	Undefined	0.108	1.563	0.462	0.696

## Data Availability

The data presented in this study are available on request from the corresponding author.
